# Reduction of neurogranin protein correlates with increases of inflammasome proteins in post mortem cases of intermediate Alzheimer’s disease

**DOI:** 10.1002/alz.089239

**Published:** 2025-01-03

**Authors:** Regina T Vontell, Ayled Barreda, Kaj Blennow, Henrik Zetterberg, Juan Pablo de Rivero Vaccari, Helen M Bramlett, W Dalton Dietrich, Robert W Keane, David A. Davis, Tatjana Rundek, Xiaoyan Sun

**Affiliations:** ^1^ University of Miami Miller School of Medicine, Miami, FL USA; ^2^ Clinical Neurochemistry Laboratory Sahlgrenska University Hospital, Mölndal Sweden; ^3^ Institute of Physiology and Neuroscience, University of Gothenburg, Mölndal Sweden; ^4^ Neurodegenerative Disorder Research Center, Institute on Aging and Brain Disorders, University of Science and Technology of China and First Affiliated Hospital of USTC, Heifei China; ^5^ Institute of Neuroscience and Physiology, Sahlgrenska Academy at the University of Gothenburg, Göteborg Sweden; ^6^ Institute of Neuroscience and Physiology Sahlgrenska Academy at the University of Gothenburg, Gothenburg Sweden; ^7^ Institute of Neuroscience and Physiology, University of Gothenburg, Mölndal Sweden; ^8^ University of Gothenburg, Gothenburg, Västergötland Sweden; ^9^ University of Gothenburg, Mölndal Sweden; ^10^ Department of Neurodegenerative Disease and UK Dementia Research Institute, UCL Institute of Neurology, Queen Square, London United Kingdom; ^11^ Department of Neurodegenerative Disease, UCL Institute of Neurology, London United Kingdom; ^12^ Hong Kong Center for Neurodegenerative Diseases, Clear Water Bay Hong Kong; ^13^ Clinical Neurochemistry Laboratory, Sahlgrenska University Hospital, Mölndal Sweden; ^14^ Department of Psychiatry and Neurochemistry, Institute of Neuroscience and Physiology, University of Gothenburg, Mölndal Sweden; ^15^ University of Gothenburg, Mölndal, Gothenburg Sweden; ^16^ Hong Kong Center for Neurodegenerative Diseases, Clear Water Bay, Hong Kong China; ^17^ The Miami Project to Cure Paralysis, Miami, FL USA; ^18^ University of Miami, Miami, FL USA; ^19^ Evelyn F. McKnight Brain Institute, Miami, FL USA; ^20^ Neurology Department, University of Miami Miller School of Medicine, Miami, FL USA

## Abstract

**Background:**

Neurogranin (Ng) is considered a biomarker for synaptic dysfunction in Alzheimer’s disease (AD). In contrast, the inflammasome complex has been shown to exacerbate AD pathology.

**Method:**

We investigated the protein expression, morphological differences of Ng and correlated Ng to hyperphosphorylated tau in the postmortem brains of 17 AD cases and 17 age and sex‐matched controls. In addition, we correlated the Ng expression with two different epitopes of apoptosis‐associated speck‐like protein containing a caspase recruitment domain (ASC).

**Result:**

We show a reduction of Ng immunopositive neurons and morphological differences in AD compared to controls. Ng immunostaining was negatively correlated with neurofibrillary tangles, humanized anti‐ASC (IC100) positive neurons and anti‐ ASC positive microglia, in AD.

**Conclusion:**

The finding of a negative correlation between Ng and ASC speck protein expression in postmortem brains of AD suggests that the activation of inflammasome/ASC speck pathway may play an important role in synaptic degeneration in AD.

